# From pilots to partnerships: Why African-Nordic collaboration can help reshape global health

**DOI:** 10.1371/journal.pgph.0006329

**Published:** 2026-05-13

**Authors:** Steven L. B. Jensen, Diane Gashumba, Anna Frellsen, Hanna Ohm Cleaver, Oscar Milsted Karstad

**Affiliations:** 1 Research Department, The Danish Institute for Human Rights, Copenhagen, Denmark; 2 Ambassador of Rwanda to the Nordics, Stockholm, Sweden; 3 Maternity Foundation, Copenhagen, Denmark; 4 Dalberg Media, Copenhagen, Denmark; 5 Danish Alliance for Global Health, Copenhagen, Denmark; PLOS: Public Library of Science, UNITED STATES OF AMERICA

Global health is entering a period of profound transition. Shifting geopolitical priorities, tightening development budgets, and growing health needs are increasing pressure on a model that has delivered important gains in recent decades. Although global health efforts have produced major results, fragmented programmes, short-term pilots, and parallel delivery structures have too often failed to build the national systems required to sustain impact at scale. The challenge is no longer primarily to identify, develop, and prove interventions, but to embed proven solutions in government-led systems that sustain delivery, build institutional capacity, and scale impact [1].

These questions were central to discussions at the inaugural African–Nordic Health Summit in Stockholm in January 2026 [2]. Convening policymakers, researchers, implementers, financiers, civil society and private sector actors from across both regions, the summit was founded on a simple premise: lasting health improvements require partnerships that move beyond isolated interventions toward integrated health systems capable of delivering impact at scale.

The summit represented not simply another global health convening, but a wider response to systemic challenges that have come to define the current global health landscape. The emerging African-Nordic partnership offers a promising model for how global health cooperation can evolve to address these challenges.

## Women’s health as a lens on system failure

The Stockholm summit deliberately focused on women’s health, including maternal health. Women account for approximately 51% of the world’s population, but more importantly, their health needs span the entire life course and cut across multiple service delivery platforms - including maternal care, primary healthcare, and prevention and management of infectious and non-communicable diseases. As such, women’s health provides one of the clearest lenses through which to understand the systemic challenges facing health systems today.

Persistent gaps in women’s health outcomes are not primarily driven by a lack of effective interventions, but by broader structural weaknesses - fragmented service delivery, insufficient workforce capacity, misaligned financing, and inconsistent political prioritization [2].

Despite falling substantially, progress has slowed and maternal mortality remains unacceptably high in many settings. This persistence of preventable maternal deaths reflects a structural failure to implement, integrate, and sustain delivery. The challenges in maternal health are not technological, but systemic, political, and financial [2,3]. Thus, conclusions regarding women’s health should be applied to global health more broadly.

Throughout the summit, participants repeatedly emphasized that the global health community has already developed many of the interventions that save lives but lack consistent delivery and scaling of what works; the processes that turn evidence into routine care. This requires workforce training, supply chains, supervision, interoperable tech platforms, and financing mechanisms to work together as an integrated whole, and to become embedded into local health systems. In other words, women’s health is not a shortage of pilots, but a shortage of systems [2].

Furthermore, these challenges are intensified by the current political landscape, anti-gender movements are gaining influence across development and human rights debates, weakening support for gender equality and related health priorities [4]. Considering tightening development finance, politically contested areas such as maternal health and sexual and reproductive health and rights are often more exposed to neglect, underfunding, or reversal [4–6].

## From donor to partner

These pressures emphasize the need to rethink how global health partnerships are structured, which was another central theme emerging from the summit. Traditional donor-recipient relationships often reinforce fragmented implementation, with external actors introducing projects that operate outside national systems. This has too often meant parallel structures, short time horizons, and weak alignment with the institutional conditions required for sustained delivery, building a growing consensus around the need to move from a donor model toward genuine partnership [1,2], echoing the recommendations of the Danish Expert Group on Global Health in December 2024 which urged Denmark to transition “from being primarily a donor to a partner in health” [7].

Equal partnerships require governments to lead in setting priorities and coordinating actors across sectors. They also require partners to align their efforts behind national systems rather than establishing parallel structures. Without this shift, progress achieved during externally funded projects often regresses once funding cycles end. Participants repeatedly highlighted this pattern throughout the summit [2].

Looking forward, especially considering the current climate, the value of these new partnership models lies in long-term capability transfer, knowledge exchange, and system strengthening, rather than in aid flows alone [2,7].

## Complementary strengths across regions

African-Nordic collaboration is fit to advance a stronger partnership model because the two regions bring complementary strengths. African countries are increasingly shaping the global health agenda through initiatives such as the African Union’s New Public Health Order, with its emphasis on regional manufacturing, health security, and greater health sovereignty [8]. Nordic countries, meanwhile, bring experience from universal healthcare systems, public governance, and integrated primary healthcare [2,7].

Bringing these strengths together creates an opportunity to build partnerships around comparative advantage rather than parallel projects. In practice, that means aligning Africa’s institutional and implementation priorities with Nordic capabilities in governance, research, innovation, and integrated care [2,7].

It also requires collaboration across sectors. Governments, development finance institutions, civil society organizations, researchers, and private sector actors need to align financing, innovation, and delivery around shared goals [2].

## Toward a new global health architecture

Global health is undergoing a structural shift toward a new architecture. At the same time, pandemic preparedness, infectious disease threats, and the growing burden of non-communicable diseases are placing new and overlapping demands on health systems [1]. Global health is being reshaped not only by changing patterns of disease, but also by political, financial, and institutional pressures.

The shift in global health partnerships is already reflected in reform efforts such as the Lusaka Agenda, which outlines a future model for global health financing and governance centered on stronger primary health care, increased domestic financing, greater equity, improved strategic coherence, and better coordination across products, research and development, and regional manufacturing [9].

In this context, African-Nordic collaboration can be seen as a practical expression of this emerging model. It represents a form of partnership designed to operate within public systems rather than around them. In an era of constrained health budgets, interventions that cannot demonstrate cost-effectiveness or align with national financing frameworks are unlikely to be sustained or scaled. This underscores the importance of domestic financing, efficiency, and accountability - and reinforces the central role of country leadership as the foundation for durable impact [2,7,9].

## Looking ahead to Kigali 2027

The African-Nordic Health Summit was conceived not as a one-off event but as the beginning of a sustained platform for collaboration. The next convening, scheduled for Kigali in 2027, aims to learn from impactful partnerships and inspire concrete initiatives across the regions [2].

Participants highlighted the importance of moving from broad commitments toward focused collaboration on a limited set of priorities where African-Nordic partnerships can demonstrate measurable progress [2]. Such partnerships could include joint initiatives to strengthen midwifery and maternal care, expand digital tools for frontline health workers, integrate non-communicable disease screening into maternal health services, and support regional health manufacturing and supply chains [2].

If successful, this new platform could offer a broader lesson for equal health partnerships: meaningful progress depends less on discovering new interventions than on building scalable platforms, systems and partnerships capable of delivering what we already know works [2].

In an era of geopolitical uncertainty and constrained resources, this shift - from fragmented pilots to equal, integrated partnerships - may be one of the most important transformations global health can make [1,2].
